# Association between the location of transposed ovary and ovarian dose in patients with cervical cancer treated with postoperative pelvic radiotherapy

**DOI:** 10.1186/s13014-019-1437-3

**Published:** 2019-12-16

**Authors:** Xiao-juan Lv, Xiao-long Cheng, Ye-qiang Tu, Ding-ding Yan, Qiu Tang

**Affiliations:** 1 Institute of Cancer and Basic Medicine (ICBM), Chinese Academy of Sciences, Hangzhou, China; 2 Department of Gynecologic Radiation Oncology, Cancer Hospital of the University of Chinese Academy of Sciences, Hangzhou, China; 3 Department of Gynecologic Radiation Oncology, Zhejiang Cancer Hospital, Bangshan East Road 1, Hangzhou, 310022 China; 4 Department of Radiation Physics, Zhejiang Cancer Hospital, Hangzhou, China

**Keywords:** Dose limit, Ovarian transposition, Ovarian function, Pelvic radiotherapy, Uterine cervical neoplasms

## Abstract

**Background and purpose:**

How to protect the ovarian function during radiotherapy is uncertain. The purpose of this study was to explore the association between the location of the transposed ovary and the ovarian dose in patients with cervical cancer received radical hysterectomy, ovarian transposition, and postoperative pelvic radiotherapy.

**Methods:**

A retrospective analysis was conducted of 150 young patients with cervical cancer who received radical hysterectomy, intraoperative ovarian transposition, and postoperative adjuvant radiotherapy in Zhejiang Cancer Hospital. Association between location of the transposed ovaries and ovarian dose was evaluated. The transposed position of ovaries with a satisfactory dose was explored using a receiver operator characteristic curve (ROC) analysis. Patients’ ovarian function was followed up 3 months and 1 year after radiotherapy.

**Results:**

A total of 32/214 (15%) transposed ovaries were higher than the upper boundary of the planning target volume (PTV). The optimum cutoff value of > 1.12 cm above the iliac crest plane was significantly associated with ovaries above the upper PTV boundary. When the ovaries were below the upper boundary of PTV, the optimum cutoff value of transverse distance > 3.265 cm between the ovary and PTV was significantly associated with ovarian max dose (Dmax) ≤ 4Gy, and the optimum cutoff value of transverse distance > 2.391 cm was significantly associated with ovarian Dmax≤5Gy. A total of 77 patients had received complete follow-up, and 56 patients (72.7%) showed preserved ovarian function 1 year after radiotherapy, which was significantly increased compared with 3 months (44.2%) after radiotherapy.

**Conclusions:**

The location of transposed ovaries in patients with cervical cancer is significantly correlated with ovarian dose in adjuvant radiotherapy. We recommend transposition of ovaries > 1.12 cm higher than the iliac crest plane to obtain ovarian location above PTV. When the transposed ovary is below the upper boundary of PTV, ovarian Dmax ≤4Gy may be obtained when the transverse distance between the ovary and PTV was > 3.265 cm, and the ovarian Dmax≤5Gy may be obtained when the transverse distance was > 2.391 cm.

## Background

Cervical cancer is the most common tumor of the female reproductive system worldwide. Approximately 30–40% of cervical cancer occurs in young women, which is an increasing trend [1]. With the progress of social economy, education, and medical technology, the survival rate of cervical cancer has been increasing yearly, and the post-treatment quality of life of patients has attracted the attention of both doctors and patients [2]. Radiotherapy is one of the main treatments for cervical cancer. The ovary is an organ that is sensitive to radiotherapy, a low dose can cause irreversible damage of the ovarian endocrine function in young patients. And then early menopause of these patients occurred which is associated with perimenopausal syndrome, including hot flashes, night sweats, irritability, and a series of lipid metabolic abnormalities, osteoporosis, as well as the corresponding complications of cardiovascular and cerebrovascular diseases [3]. Therefor, preservation of ovarian endocrine function is important to quality of life for the young patients with cervical cancer.

A total of 0.19–1.3% of cervical squamous cell carcinomas metastasize to the ovary, and metastasis rate of adenocarcinoma is 1.4–8.2% [4–6]. Therefore, ovarian preservation in patients with cervical squamous cell carcinoma is recognized as safe and feasible, whereas in other pathological types it remains controversial. The ovary is located in the pelvis and is damaged by postoperative pelvic radiotherapy [7]. McCall et al. first described the ovary transposition out of the pelvic radiation fields as an effective method to avoid radiation injury of ovaries during postoperative radiotherapy in 1958 [8]. Subsequently, ovarian transposition has been increasingly common to protect ovarian function in young patients with early cervical squamous cell carcinoma [5, 6, 9]. However, several reports have indicated that only approximately 50% of the transposed ovarian function can be preserved for patients treated with pelvic radiotherapy, whereas ovarian failure has been observed in other patients [5, 10, 11].

The preservation of the ovarian endocrine function in patients with cervical cancer after radiotherapy is directly associated with ovarian dose [12, 13]. The ovarian dose received in pelvic radiotherapy might be related to the position of the transposed ovary. However, few studies have explored the relationship between ovarian transposition and ovarian dose. Currently, whether the ovary needs to be transposed to successfully retain ovarian function is still uncertain. Therefore, we designed the present study to explore the association between the location of the transposed ovary and ovarian dose in patients with cervical cancer who underwent radical hysterectomy, ovarian transposition, and postoperative pelvic radiotherapy. Furthermore, we sought to predict the position of ovarian transposition to obtain a satisfactory radiation dose of the ovary in postoperative pelvic radiotherapy.

## Methods

### Patients

A retrospective analysis was conducted of 150 young patients with cervical cancer who underwent radical hysterectomy, intraoperative ovarian transposition and postoperative adjuvant radiotherapy in Zhejiang Cancer Hospital (Hangzhou, Zhejiang, China) from January 2011 to June 2017. This study was approved by the Ethics Committee of the Zhejiang Cancer Hospital and the subject gave informed consent. All cervical cancer patients received standard radical hysterectomy and unilateral or bilateral ovarian transposition at the peritoneum of the paracolic sulci. All patients were ≤ 45 years old (the youngest was 22 years old), with an average age of 35.8 ± 5.24 years. According to the International Federation of Gynecology and Obstetrics (FIGO) staging criteria in 2009 [14], 77 (51.3%) patients were stage IB1, 39 (26%) stage IB2, 13 (8.7%) stage IIA1, 13 (8.7%) stage IIA2, six (4%) stage IIB, and two (1.3%) had residual recurrence. Most of the cases were squamous cell carcinoma (140 cases, 93.3%), followed by adenocarcinoma (five cases, 3.3%), adenosquamous carcinoma (four cases, 2.7%), and neuroendocrine carcinoma (one case, 0.7%). All patients received external pelvic radiotherapy, and two patients with residual recurrence received external pelvic and vaginal intracavitary radiotherapy. External pelvic radiotherapy included three-dimensional conformal radiotherapy (3D-CRT) and intensity-modulated radiotherapy (IMRT).

### Radiotherapy

The body membrane was first established for CT simulation and treatment of each patient. The Philips BrillianceTM16 row CT simulation machine with large aperture was used to simulate the location of all patients with postoperative adjuvant external radiotherapy. CT images were acquired using 5-mm-thick contiguous slices with a radiotherapy CT simulator (Philips Brilliance Big Bore) and transferred to the treatment planning system (Philips Pinnacle^3^ TPS). The clinical target volume (CTV) covered the common iliac, external iliac, internal iliac, obturator, presacral and parametrial lymph node regions, and was delineated according to the guidelines defining the pelvic node CTV in external beam radiotherapy for uterine cervical cancer of radiation therapy oncology group (RTOG) [15]. The planned target volume (PTV) was generated by uniformly expanding the CTV boundary by 0.7 cm in three dimensions. Organs at risk (OAR) including bladder, rectum, bowel bag, spinal cord, bone marrow and ovaries were contoured according to the recommendations of the International Commission on Radiation Units Reports (ICRU) 50 and 62 [16, 17]. All radiotherapy plans were created by the physicists with the PTV prescription of 45–50Gy / 25–28F, and the Dmax of the ovaries was ≤6Gy. A total of 59/150 (39.3%) patients received 3D-CRT, and 91/150 (60.7%) patients received IMRT.

### Observations

**Fig. 1:**
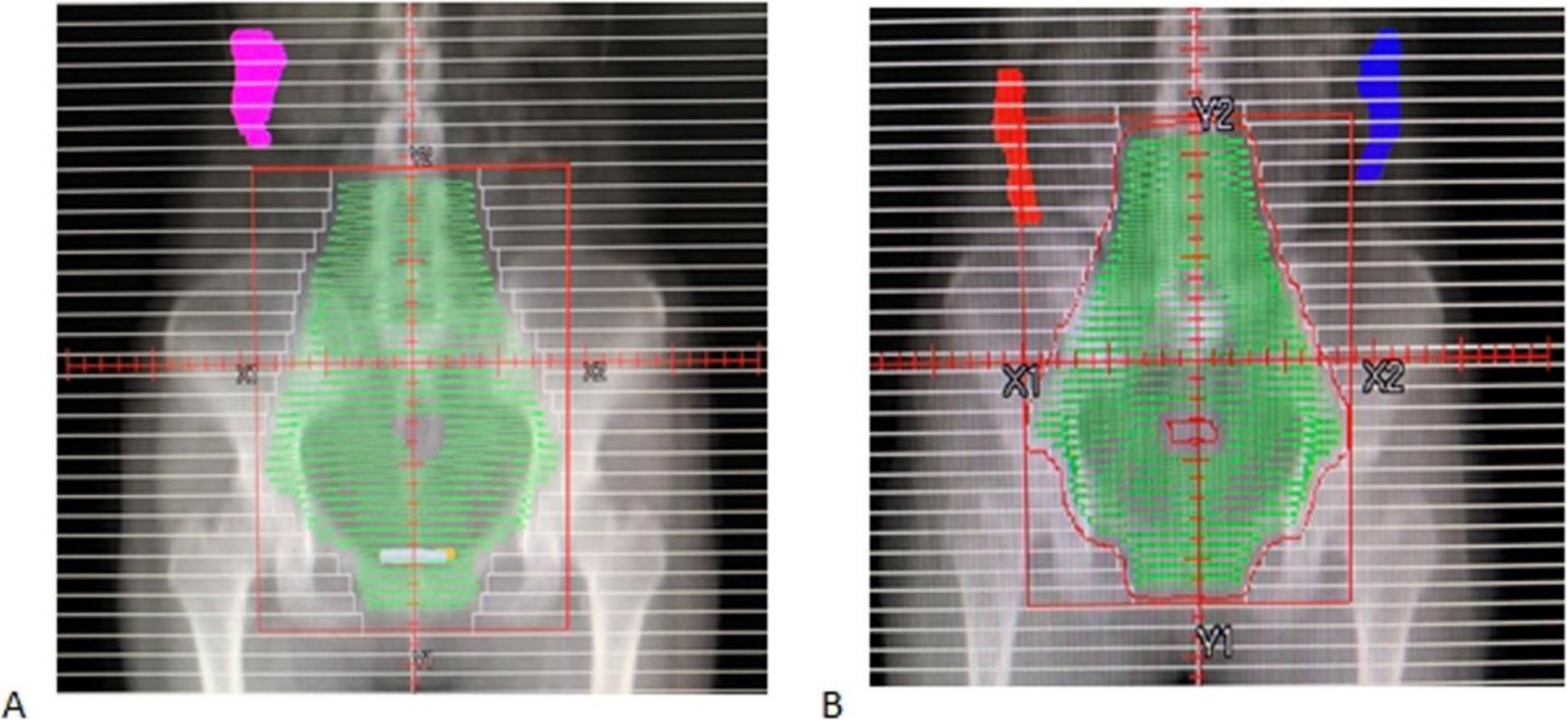
Relationship between ovary position and PTV. **a** Lower boundary of the ovary is above the upper boundary of the PTV. **b** Lower boundary of the ovary is below the upper boundary of the PTV

Records were reviewed for patients’ age, height, weight, body mass index (BMI), clinical stage, pathological type, ovarian volume, ovarian cysts, side of ovarian transposition, method of radiotherapy, position of transposed ovary, and the Dmax of ovary. The position of the transposed ovary was measured as three distances including the vertical distance between the lower boundary of the ovary and the iliac crest plane, the vertical distance between the lower boundary of ovary and the upper boundary of the PTV, and the transverse distance between the medial boundary of the ovary and the lateral boundary of the PTV. The vertical distance was a negative value when the lower boundary of the ovary was below the iliac crest plane or below the upper boundary of the PTV. Based on the vertical distance between the lower boundary of the ovary and the upper boundary of the PTV, the patients were divided into two groups, including upper (the lower boundary of the ovary was above the upper boundary of the PTV) and lower (the lower boundary of the ovary was under the upper boundary of PTV) (Fig. 1). The observations of the two groups were compared and analyzed to explore the optimal location of the transposed ovary to be above the PTV. Ovaries which were partially or totally completely below the upper boundary of PTV and received dose limit were subdivided into two groups, including satisfactory (Dmax ≤400 cGy) and unsatisfactory (Dmax > 400 cGy). The observations of two groups were compared. The ROC curve was drawn to predict the location of the transposed ovary, where the ovary Dmax ≤400 cGy and the ovary Dmax ≤500 cGy could be obtained.

### Follow-up

During the follow-up, the ovarian endocrine function of 150 patients were evaluated 3 months after radiotherapy and 1 year after radiotherapy. The sex hormone levels, including estrogen [E2], follicle stimulating hormone [FSH], and luteinizing hormone [LH] were measured. Simultaneously, menopausal symptoms of the patients were assessed. Ovarian function was considered normal when the FSH was < 40 mIU/mL and E2 > 50 pg/mL, and the patients showed no menopausal symptoms.

### Statistical analysis

Mean values between the groups were compared using the Student’s *t* test. Frequency data between the groups were compared using the χ2 test. A binary logistic regression model was used to determine the independent risk factors for satisfactory ovarian dose (yes vs. no). The receiver operating characteristic curve (ROC curve) was drawn to predict the location of the transposed ovary, where the ovary can above PTV and a satisfactory ovarian dose could be obtained. The data were analyzed by SPSS software (Statistical Package for the Social Sciences, SPSS, Version 19.0, Chicago, IL). A value of *P* < 0.05 was considered statistically significant.

## Results

A total number of 214 transposed ovaries were analyzed from 150 patients. Of the 150 patients, 78 (52%) had a right ovarian transposition, 64 (42.7%) had a bilateral ovarian transposition, and eight (5.3%) had a left ovarian transposition. Out of the 214 transposed ovaries, there were 143 (66.8%) on the right side and 71 (33.2%) on the left side. The lower boundary of all transposed ovaries was 0.61 ± 2.64 cm from the iliac crest plane. The lower boundary of the transposed ovary above the iliac crest plane was observed in 121 (56.5%) cases, whereas the lower boundary of the ovary of 93 (43.5%) cases was below the iliac crest plane. The distance between the lower boundary of the transposed ovary and the iliac crest plane showed a significant difference between the right and left ovaries (1.05 ± 2.64 cm vs. -0.27 ± 2.44 cm, *t* = 3.551, *P* = 0.000). There were 40/214 (18.7%) ovarian cysts, and no ovarian metastases were observed.

**Fig. 2:**
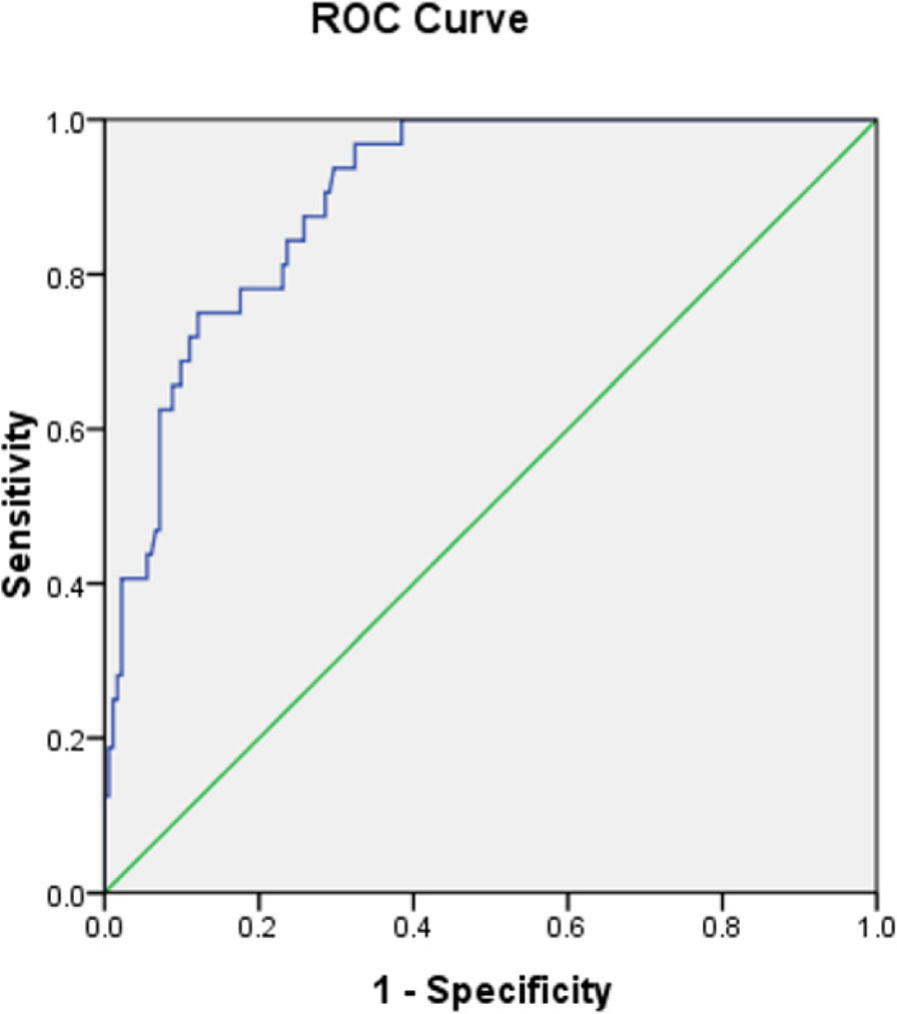
ROC curve of the distance between the ovary and iliac crest plane to predict ovary above PTV. The AUC was 0.899 (95% CI: 0.853–0.946, *p* = 0.000), and the optimal cut-off point value was 1.12 cm

Out of the 214 transposed ovaries, 32 (15%) were above PTV, and the Dmax was ≤400 cGy, which was a satisfactory dose; 182 (85%) transposed ovaries were partially or completely below the upper boundary of the PTV. Among these 182 ovaries, the radiation dose limit was abandoned in 38 because the ovaries were too close to the radiation fields, and the remaining 144 ovaries received the dose limit. We observed a significant difference in the distance between the ovary and the iliac crest plane, the radiotherapy method and the ovarian Dmax between the two groups (*P* < 0.05), as shown in Table 1. The ROC curve was drawn, which is presented in Fig. 2. The distance between the transposed ovary and the iliac crest plane was significant for predicting the ovarian location above the PTV. The area under the curve [AUC] was 0.899 (95% confidence interval [CI]: 0.853–0.946, *P* = 0.000). The optimal cut-off point value was 1.12 cm, the sensitivity was 0.969, the specificity was 0.676, and the Youden index was 0.645.

**Table 1:** Characteristics of patients with the ovary above or below PTV

Characteristics	Above PTV (*n* = 32)	Below PTV (*n* = 144)	*P-*value
Ovarian volume, cm3	12.84 ± 7.92	13.63 ± 9.13	0.650^*^
Ovarian cyst, case (rate)	6 (18.8%)	29 (20.1%)	0.859^#^
Side of transposed ovaries			0.117^#^
Right side	27 (84.4%)	102 (70.8%)	
Left side	5 (15.6%)	42 (29.2%)	
Radiotherapy methods			0.001^#^
3D-CRT	21 (65.6%)	47 (32.6%)	
IMRT	11 (34.4%)	97 (67.4%)	
Distance from iliac crest plane, cm	3.87 ± 1.92	0.49 ± 2.20	0.000^*^
Dmax≤400 cGy, case (rate)	32 (100%)	57 (39.6%)	0.000^#^

^*^Student’s *t*-test

^#^ *χ*^2^ test

A total of 144 ovaries which were partially or completely below the upper boundary of PTV and received dose limit were subdivided into two groups according to ovarian Dmax(≤400 cGy or >400 cGy). Statistical analysis showed significant differences in the distances between the ovaries and the iliac crest plane as well as the transverse distance between the ovary and the PTV between the two Dmax level groups (*p* < 0.01), as can be seen in Table 2.

**Table 2:** Characteristics of patients with the ovarian Dmax greater or less than 400 cGy

Characteristics	Dmax *≤*400 cGy (*n* = 57)	Dmax > 400 cGy (*n* = 87)	*P-*value
Height, cm	158.58 ± 5.72	159.29 ± 4.47	0.407^*^
Weight, kg	56.15 ± 10.69	54.74 ± 9.82	0.638^*^
BMI	22.06 ± 3.85	21.57 ± 3.69	0.445^*^
Ovarian volume, cm3	12.45 ± 8.12	14.40 ± 9.69	0.209^*^
Ovarian cyst, case (rate)	11 (19.3%)	18 (20.7%)	0.839^#^
Side of transposed ovaries			0.083^#^
Right side	45 (78.9%)	57 (65.5%)	
Left side	12 (21.1%)	30 (34.5%)	
Radiotherapy methods			0.190^#^
3D-CRT	15 (26.3%)	32 (36.8%)	
IMRT	42 (73.7%)	55 (63.2%)	
Distance from iliac crest plane, cm	1.08 ± 2.28	0.10 ± 2.06	0.008^*^
Transverse distance from PTV, cm	4.03 ± 1.15	2.88 ± 1.01	0.000^*^
Longitudinal distance from PTV, cm	−3.22 ± 2.41	−3.89 ± 2.12	.083^*^

^*^Student’s *t*-test

^#^ *χ*^2^ test

**Fig. 3:**
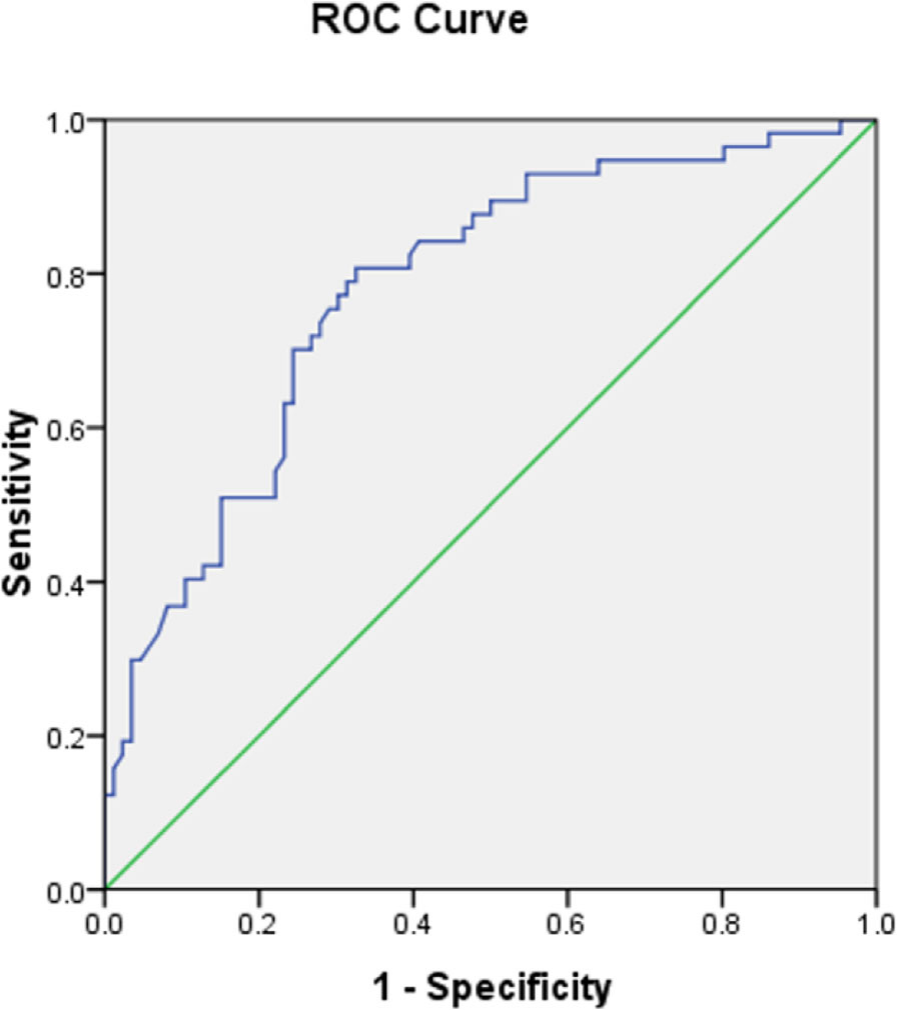
ROC curve of the distance between the ovary and PTV to predict the ovarian Dmax ≤400 cGy. The AUC was 0.779 (95% CI: 0.703–0.856, *p* = 0.000), and the optimal cut-off point value was 3.265 cm

**Fig. 4:**
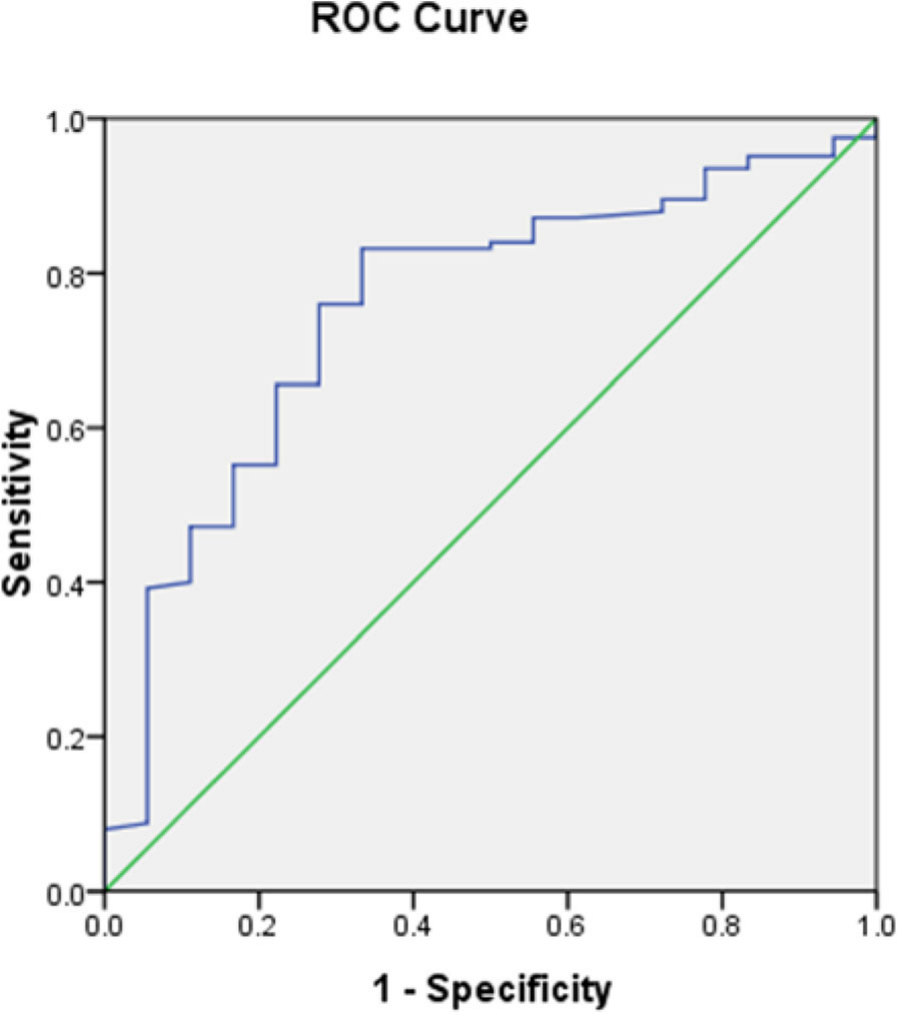
ROC curve of the distance between the ovary and PTV to predict the ovarian Dmax ≤500 cGy. The AUC was 0.755 (95% CI: 0.638–0.872, *p* = 0.000), and the optimal cut-off point value was 2.391 cm

Logistic multivariate regression analysis showed that the transverse distance between the ovary and PTV was an independent correlative factor to predict the ovarian Dmax≤400 cGy during the radiotherapy, as shown in Table 3. The ROC curve was drawn, which is presented in Fig. 3. The transverse distance between the ovary and PTV was significant for predicting the ovarian Dmax≤400 cGy. The AUC was 0.779 (95% CI: 0.703–0.856, *p* = 0.000). The optimal cut-off point value was 3.265 cm, the sensitivity was 0.807, the specificity was 0.674, and the Youden index was 0.481. The transverse distance between the ovary and PTV was also significant for predicting the satisfaction of ovarian Dmax≤500 cGy. The AUC was 0.755 (95% CI: 0.638–0.872, *p* = 0.000). The optimal cut-off point value was 2.391 cm, the sensitivity was 0.832, the specificity was 0.667, and the Youden index was 0.499, as shown in Fig. 4.

**Table 3:** Independent risk factors to predict the satisfactory ovarian dose of radiotherapy

Characteristics	OR	95%CI	*P-*value
Distance from iliac crest plane	0.947	0.760–1.181	0.631
Transverse distance from PTV	0.377	0.248–0.571	0.000^&^
Longitudinal distance from PTV	1.002	0.807–1.243	0.985

^&^*P* < 0.05

**Fig. 5:**
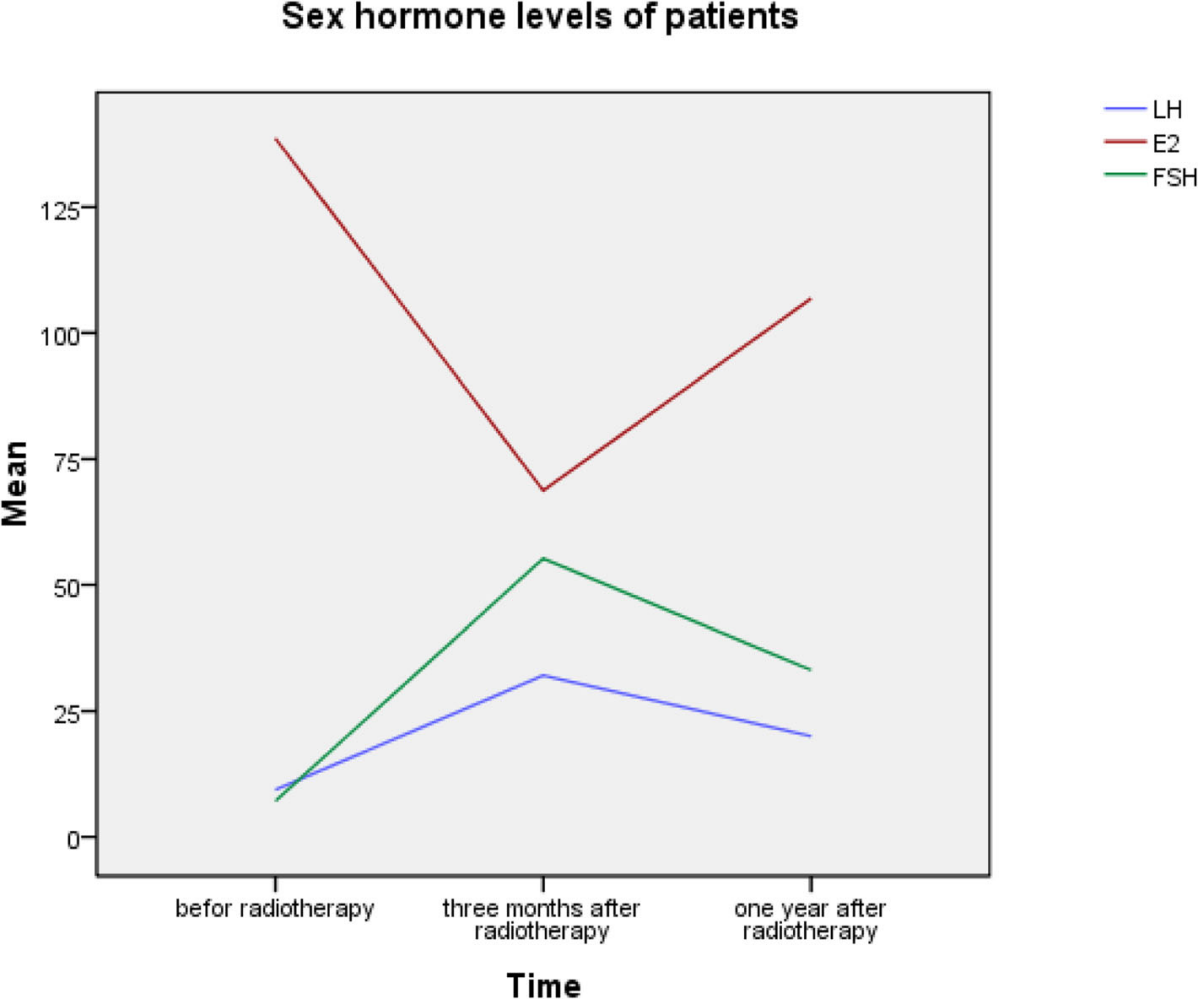
Sex hormone levels at different times points pre- and post-radiotherapy. The E2 levels were significantly decreased 3 months post-radiotherapy, but rebounded one-year post-radiotherapy. The FSH and LH levels were opposite trends

The ovarian endocrine function of 150 patients was evaluated 3 months and one-year post-radiotherapy, and 77/150 patients reached complete followed-up. The sex hormones in the 77 patients were normal pre-radiotherapy. The E2 levels in the patients were significantly decreased 3 months post-radiotherapy, but significantly rebounded one-year post-radiotherapy. Conversely, the FSH and LH levels were significantly increased 3 months post-radiotherapy and were significantly decreased one-year post-radiotherapy, and the differences were statistically significant (Fig. 5, Table 4). According to FSH levels, E2 serum, and menopausal symptoms, 34/77 patients (44.2%) had normal ovarian function 3 months post-radiotherapy. However, 7/34 patients lost ovarian function one-year post-radiotherapy, and 29 of the remaining 43 patients regained ovarian endocrine function one-year post-radiotherapy. Therefore, 56/77 (72.7%) patients had preserved ovarian function one-year post-radiotherapy, which was significantly higher than 3 months post-radiotherapy (*p* = 0.000; Table 4).

**Table 4:** Sex hormone levels and ovarian function in patients pre- and post-radiotherapy

Characteristics	Pre-radiotherapy	Three months post-radiotherapy	One-year post-radiotherapy
E2, pg/mL	138.68 ± 90.91	68.75 ± 98.77^*^	106.85 ± 126.46^&^
FSH, mIU/mL	7.13 ± 6.40	55.25 ± 43.35^*^	33.14 ± 40.58^&^
LH, mIU/mL	9.35 ± 9.67	32.09 ± 24.49^*^	20.02 ± 23.62^&^
Normal ovarian function, case (rate)	77(100%)	34 (44.3%)	56(72.7%) ^#^

^*^Student’s *t*-test, Comparison of sex hormone levels with pre-radiotherapy. *P* < 0.01

^&^Student’s *t*-test, Comparison of sex hormone levels with 3 months post-radiotherapy. *P* < 0.05

^#^*χ*^2^ test, Comparison of cases of normal ovarian function with 3 months post-radiotherapy. *P* < 0.01

## Discussion

Cervical cancer is a non-hormone dependent tumor, and the probability of early cervical cancer metastasis to the ovary is extremely low. Yamamoto et al. [18] reported ovarian metastasis in 2/485 (0.4%) cases of early cervical squamous cell carcinoma and ovarian metastasis in 12/146 (8.2%) cases of non-squamous cervical cancer. Additionally, Shimada et al. [19] found that ovarian metastasis in 52/3471 (1.5%) patients with stage Ib-IIb cervical cancer, and that the metastasis rate of cervical adenocarcinoma was significantly higher than cervical squamous cell carcinoma (5.31% vs. 0.79%). Landoni et al. [20] found ovarian metastasis in 16/1695 (0.9%) cases of Ia2-IIa cervical cancer with bilateral ovariotomy. Therefore, it is safe and feasible to retain ovaries in patients with early cervical squamous cell carcinoma. In the present study, the incidence of ovarian cysts was 18.7%, and no case of translocated ovarian metastasis was found. In addition, the right ovary transposition was significantly higher than the left ovary, and the distance between the right ovary and the iliac crest plane was significantly higher than the left ovary. We speculated that this finding was related to the position of the surgeon, possibly the surgeon and staff were attending to the patient on the left side.

The rate of preserving the translocated ovarian endocrine function after pelvic radiotherapy was approximately 50%, and the dose received by the ovary during radiotherapy and the age of the patients were two important influencing factors [5, 10, 11]. At the time of our study, there was no guideline or recommendation on the dose limit of radiotherapy in the ovary. Swerdlow et al. [21] found that the risk of premature ovarian failure for women under 40 years old was significantly increased when their ovaries received a radiation dose higher than 5 Gy. Husseinzadeh et al. [12] considered that the ovarian dose above 3 Gy significantly increased the risk of premature ovarian failure. Wallace et al. [13] found that a single dose of more than 8 Gy or a fractionated dose of more than 15 Gy caused a permanent damage of the ovarian function in most all women, whereas a dose lower than 1.5 Gy result in a minor effect on ovarian function. A follow-up study found that 64% of patients who received a radiation dose of less than 5 Gy after bilateral ovarian transposition retained their ovarian function [22]. Therefore, in our study, we considered a value of ovarian Dmax lower than 400 cGy to be a satisfactory dose.

The preservation of ovarian function has been directly related to its translocated position [23]. Charmber et al. [24] reported that 59–100% of patients would lost ovarian function when the ovary was transposed below the iliac crest, and 70–90% of patients would preserve the ovarian function when the transposed ovary above the iliac crest. Therefore, the ovary was recommended to be transposed over 1.5 cm above the iliac crest. Toman et al. [25] observed the endocrine function of transposed ovaries after external pelvic radiotherapy and found that 2.5 cm away from the edge of the radiation field was a safe area. Hwang et al. [9] reported that a distance greater than 1.5 cm between the transposed ovary and the iliac crest plane was an independent prognostic factor for the preservation of ovarian endocrine function. However there have been few studies reported to determine the relationship between the transposed position and the ovarian dose in radiotherapy. Yoshihiro et al. [26] used volumetric modulated arc therapy (VMAT) with limited angle to make plans and found that the average ovarian dose could be reduced to below 300 cGy only when the transverse distance between the transposed ovaries and PTV was greater than 6.1 cm.

In the present study, we found that the position of the transposed ovaries was significantly correlated with the ovarian dose in radiotherapy. When the transposed ovaries were above the PTV, a satisfactory dose could be obtained by IMRT or 3D-CRT. The ROC curve showed that the distance between the transposed ovaries and the iliac crest plane was significant for predicting the ovary above PTV, and the optimal cutoff value was 1.12 cm. When the lower boundary of transposed ovaries below the upper boundary of the PTV, we found that the transverse distance between the ovary and PTV was significance for predicting the ovarian Dmax. For these patients, IMRT is recommended to reduce the ovarian radiation dose to a satisfactory level.

When the ovarian function is damaged by radiation, E2 secretion will be significantly reduced, whereas FSH and LH levels will increase, leading to perimenopausal symptoms, such as hot flashes and night sweats. The percentage of patients with normal ovarian function after radiotherapy varies widely. Buekers TE. et al. [11] analyzed 80 patients with cervical cancer who had received a radical hysterectomy and ovarian transposition and 26/80 received postoperative adjuvant radiotherapy, and found that only 42% of patients with postoperative adjuvant radiotherapy retained ovarian endocrine function at long-term follow-up. Pahisa J et al. [27] found that ovarian endocrine function was retained in 63.6% of patients with cervical cancer who received adjuvant radiotherapy after ovarian transposition, whereas ovarian function was retained in 93% of patients who did not receive adjuvant radiotherapy. AI-Badawi et al. [28] found that 65% of patients with cervical and rectal cancer who underwent ovarian transposition had successfully preserved ovarian endocrine function after a 33-month follow-up. Yin lina et al. [29] showed that 41/105 (39.0%) patients who underwent IMRT with a limited radiation dose to the ovaries preserved their normal ovarian function. In the present study, 34 patients (44.2%) exhibited normal ovarian function at 3 months after radiotherapy, however 56 patients (72.7%) showed normal ovarian function at 1 year after radiotherapy. Interestingly, the average E2 level of the patients was the lowest 3 months after radiotherapy and gradually increased at one-year post-radiotherapy. Approximately 67% of patients who had lost ovarian endocrine function 3 months after radiotherapy regained it one-year post-radiotherapy. This finding could be related to the different state of the follicles in the ovary, and the mature follicles are more sensitive to radiation than the primordial follicles [30]. When the ovaries receive low doses of radiation, the mature ovarian follicles with hormone-secreting function are first to die, whereas the primordial follicles survive. Following a long period of growth and development of into mature follicles, the ovary will likely secrete sex hormones again.

## Conclusions

The location of the transposed ovary in patients with cervical cancer was significantly correlated with the ovarian dose of postoperative adjuvant radiotherapy. Transposition of the ovary higher than 1.12 cm above the iliac crest plane was recommended to obtain ovarian location above PTV. However, if the lower boundary of the transposed ovary is below the upper PTV boundary, the ovarian Dmax≤400 cGy may be obtained when the transverse distance between the ovary and PTV was > 3.265 cm, and ovarian Dmax≤5Gy may be obtained when the transverse distance was > 2.391 cm using IMRT.

## Data Availability

The datasets used and analyzed during the current study are available from the corresponding author on reasonable request.

## Abbreviations

3D-CRT: Three dimensional conformal radiotherapy
AUC: Area under curve
CI: Confidence interval
CTV: Clinical target volume
IMRT: Intensity modulated radiation therapy
OAR: Organ at risk
PTV: Planning target volume
ROC: Receiver operating characteristic curve
Se: Sensitivity
Sp: Specificity
YI: Youden index
